# Pearls and pitfalls for comminuted distal radius fractures

**DOI:** 10.1186/1753-6561-9-S3-A34

**Published:** 2015-05-19

**Authors:** Alejandro Badia

**Affiliations:** 1 Badia Hand to Shoulder Center, Miami, Florida, 33166, USA

## 

Fractures of the distal end of radius have been estimated to account for nearly 20% of all fractures seen in a routine emergency room. Despite this, until very recently, the distal radius fracture was not treated as aggressively as other less common peri-articular fractures. Perhaps Abraham Colles’ perception that this fracture had good outcomes, no matter what was done had permeated our thinking. The reality is that frequent poor outcomes in wrist injury fuelled a gradual, albeit delayed, pursuit of a better treatment option.

Distal radius fractures are usually sustained by elderly osteoporotic patients after a fall or by younger patients as a result of high energy trauma. Both of these fractures deserve stable internal fixation, the former due to osteoporotic bone that demands sound fixation principles and the latter requires the ability to reduce intra-articular comminuted fragments in a stable manner that maintains the reduction and permits early mobilization. Until the advent of volar fixed angle plating, no technique could satisfy these requirements in a consistent manner.

Since the first description of Colles fracture in 1814, the treatment options for distal radius fractures have varied from relative neglect to active management by open reduction and internal fixation with combined approaches. In the past decade, distal radial fixation has benefited from the limelight as different groups of investigators have been developing new implants and techniques to improve the outcome of the fractures. Nevertheless, unstable fractures of the distal radius still remain challenging problems for orthopedic surgeons with hand surgeons taking the lead to alter the basic premise of how these should be managed. The consensus prevails that the majority of displaced distal radial fractures are articular injuries resulting in the disruption of both the radiocarpal and the distal radioulnar joint, hence, demanding more precise reduction.

The importance of achieving and maintaining both an anatomic reduction of the articular surface and the extraarticular alignment of the distal radius has been documented by several clinical and biomechanical studies.

Furthermore, the principle of treatment for distal radial fracture is the same for any other articular fracture i.e. articular reconstruction, stable fixation and early motion. However, in articular fractures, particularly with comminution, certain technical pearls are very helpful for restoring the anatomy and congruity:

1. An experienced assistant is invaluable in maintaining longitudinal traction and provisional reduction while the plate is initially placed.

2. The surgical approach must allow for tension free visualization of the volar margin and watershed line of the distal radius. My particular preference is to make a Bruner type extension of the volar incision as the crease lines are crossed.

3. The first compartment should be released and brachioradialis elevated subperiostially to allow for regaining the length of the radial column.

4. Die-punch fragments or severe comminution can be approached intrafocally by pronating the shaft out of the wound via the extended FCR approach.

5. Subchondral K-wires may assist in maintaining the reduction while a single subchondral distal locking screw is placed in order to gauge the reduction and plate placement during initial fluoroscopic evaluation.

6. If reduction suffices, the remainder of the screws are placed and one can then check stability under life fluoroscopy.

7. In either high energy injuries or younger patients with high demand activities, I generally recommend arthroscopic evaluation of the reduction and assessment for any critical articular soft tissue injuries that can then be addressed at that time.

Adhering to these critical pearls may help in avoiding the pitfalls of treating comminuted distal radius fractures , maintaining the same attention to detail as any weight-bearing joint sustaining a severe articular injury.

